# Promotoras (community health workers) improve heart health among Latinos in rural and urban settings

**DOI:** 10.1186/1748-5908-10-S1-A55

**Published:** 2015-08-20

**Authors:** Katrina Kubicek, Marisela Robles, Lourdes Baezconde-Garbanati, Melinda Cordero-Barazaga, Michele D Kipke

**Affiliations:** 1 Community Engagement Program, Southern California Clinical and Translational Science Institute, University of Southern California, Los Angeles, CA, 90089, USA; 2 Department of Preventive Medicine, Keck School of Medicine, University of Southern California, Los Angeles, CA, 90089, USA; 3 Vision y Compromiso, Los Angeles, CA, 90012, USA; 4 Division of Research on Children, Youth and Families, Children's Hospital Los Angeles, Los Angeles, CA, 90027, USA

## 

The project was aimed at reducing cardiovascular health disparities by implementing an evidence-based curriculum (Su Corazón Su Vida) delivered by promotoras in underserved communities. This 11-session intervention was designed to promote heart health through interactive exercises and promoting healthy lifestyle choices.

## Objectives

Twenty-five promotoras were trained in the curriculum; they in turn reached 750 Latino participants through community workshops. In order to reach Latinos in both urban and rural settings, we focused on both rural (Kern County) and urban (Los Angeles) communities in Southern California.

## Methods

A community-engaged research approach was taken as it involved the collaboration of a research institution and a community-based organization. Promotoras obtained human subjects certification, were trained on research protocols, obtained informed consent, collected de-identified pre and post data from community participants, and contributed to data interpretation as community advisory board members.

## Results

Compared to baseline, 3-month follow-up data indicate community participants made significant changes in: 1) the amount of physical activity performed (p < 0.001); 2) consumption of fruits and vegetables (p < 0.001); and 4) the proportion who implemented lifestyle changes in their households to decrease their risk for cardiovascular disease (p < 0.001).

## Discussion

This project adds to the evidence-base that promotoras can be important players in the field of dissemination and implementation science while also ensuring cultural competency - important when considering health disparities. As trusted and known members of the community, protmotoras can effectively recruit and retain community members into evidence-based programs. Also, promotoras can be active partners in research with appropriate training and supervision. A 12-month follow-up with community participants is currently underway.

## Funding source

This project was supported through a contract with the Altarum Institute and a grant from the National Institute of Heart, Lung and Blood Institute (GS10F0231K/HHSN268200900114U).

